# Optimization of fluorophores for chemical tagging and immunohistochemistry of Drosophila neurons

**DOI:** 10.1371/journal.pone.0200759

**Published:** 2018-08-15

**Authors:** Geoffrey W. Meissner, Jonathan B. Grimm, Rebecca M. Johnston, Ben Sutcliffe, Julian Ng, Gregory S. X. E. Jefferis, Sebastian Cachero, Luke D. Lavis, Oz Malkesman

**Affiliations:** 1 Janelia Research Campus, Howard Hughes Medical Institute, Ashburn, Virginia, United States of America; 2 Division of Neurobiology, Medical Research Council Laboratory of Molecular Biology, Cambridge, United Kingdom; Imperial College London, UNITED KINGDOM

## Abstract

The use of genetically encoded ‘self-labeling tags’ with chemical fluorophore ligands enables rapid labeling of specific cells in neural tissue. To improve the chemical tagging of neurons, we synthesized and evaluated new fluorophore ligands based on Cy, Janelia Fluor, Alexa Fluor, and ATTO dyes and tested these with recently improved *Drosophila melanogaster* transgenes. We found that tissue clearing and mounting in DPX substantially improves signal quality when combined with specific non-cyanine fluorophores. We compared and combined this labeling technique with standard immunohistochemistry in the Drosophila brain.

## Introduction

Immunohistochemistry (IHC) allows the visualization of specific antigens in tissue using the binding of fluorophore-labeled antibodies. Although IHC is a relatively simple technique and has been widely used for decades [1,2], this method has well-known limitations [3,4] including poor tissue penetrance of the antibodies, high background staining (e.g. [5]), and cross-reactivity between antibodies (e.g. [6]). Poor tissue penetrance of antibodies often lengthens the time required for IHC protocols. Non-specific binding of antibodies causes high background staining that masks the detection of the target antigen. Cross-reactivity can occur when antibodies developed against a protein in one species bind to related proteins in another species. Collectively, these issues make IHC time consuming and difficult to optimize.

The self-labeling tag concept offers an alternative method to label structures of interest in tissue. Instead of a relatively large antibody binding to an epitope, a small molecule ligand covalently binds to a genetically encoded enzyme-based “tag” (*e*.*g*., HaloTag, SNAP-tag) expressed in a specific cellular location [7–9]. Originally developed as a complement to fluorescent proteins in live cell fluorescence microscopy, chemical tags have been adapted for use in fixed tissue. The small size of the chemical tag ligand allows rapid labeling in tissue as well as lower background staining and cross-reactivity [10]. However, although the initial chemical tag reporters established the viability of chemical labeling of Drosophila fixed tissue, their labeling intensity was low. The second-generation reporters substantially increased labeling intensity, as well as expanding the range of expression systems that could be used with chemical tags [11]. Therefore, these new reporters offer a more potent method for rapid fixed tissue labeling while avoiding the limitations of the traditional IHC approach mentioned above.

**Fig 1:**
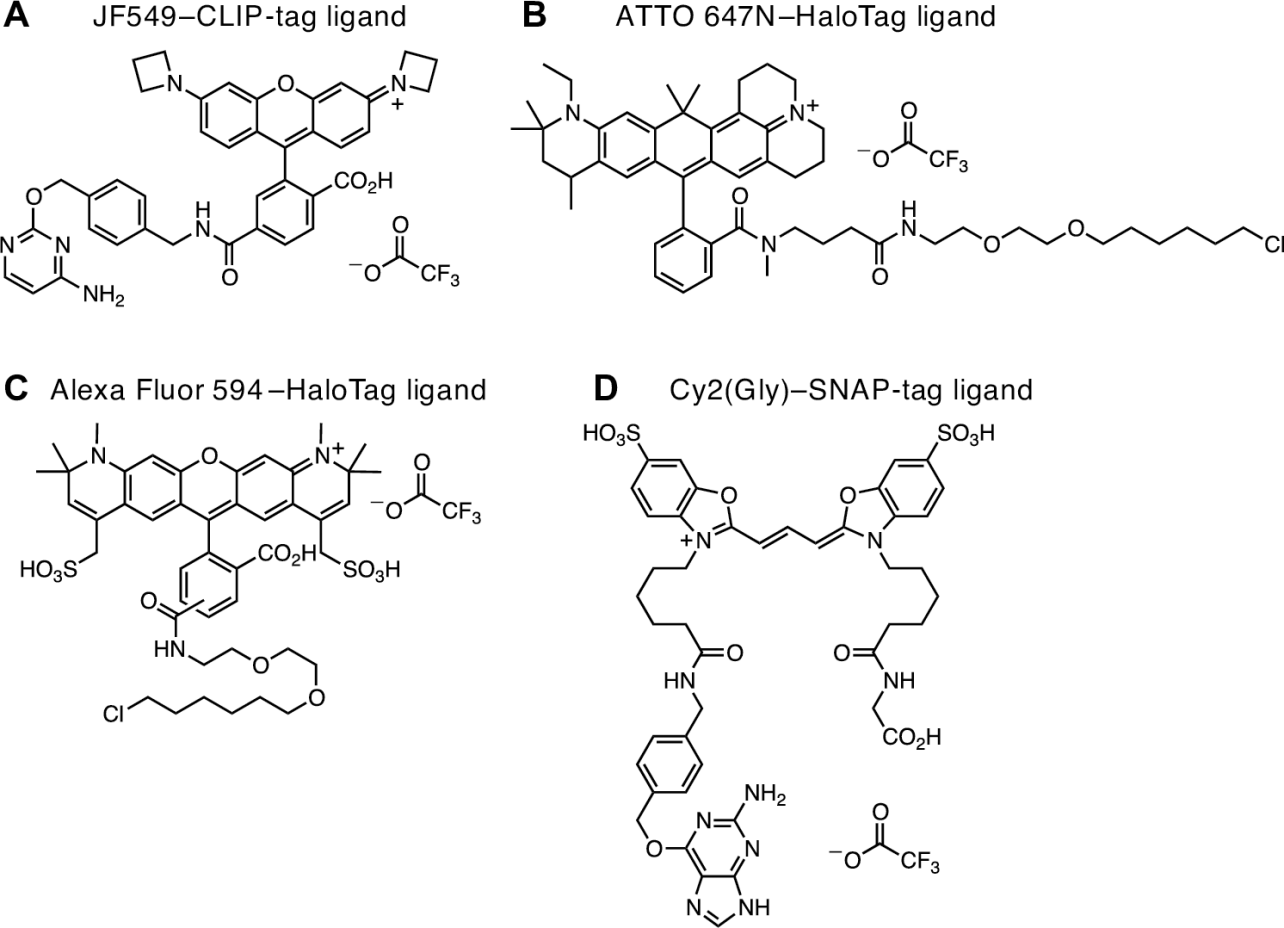
Chemical tag ligands. (A) JF_549_–CLIP-tag ligand. (B) ATTO 647N–HaloTag ligand. (C) Alexa Fluor 594–HaloTag ligand. (D) Cy2(Gly)–SNAP-tag ligand.

Despite the utility of chemical tagging in tissue, the existing collection of commercial fluorophore ligands was not developed explicitly for fixed tissue labeling. Here, we evaluate chemical tagging to replace or complement IHC labeling of neurons in the *Drosophila melanogaster* central nervous system. We used the recently developed expression systems for genetically encoded tags [10,11], as well as designed and synthesized four new chemical tag ligands: Cy2 SNAP-tag, JF_549_ CLIP-tag, Alexa Fluor 594 HaloTag, and ATTO 647N HaloTag (**Fig 1**; [12]). In particular, we investigated the performance of these dyes in conjunction with xylene tissue clearing and DPX (Distyrene, Plasticizer, and Xylene) mounting medium to match the refractive index of glass [13].

## Methods

### Chemical synthesis

Commercial reagents and solvents were obtained from Sigma-Aldrich or Fisher Scientific unless otherwise noted. BG-NH_2_ and BC-NH_2_ were acquired from New England Biolabs (NEB), and HaloTag amine (O2) ligand was purchased from Promega. ATTO 674N NHS ester, Alexa Fluor 594 NHS ester, and Cy2 bis-NHS ester were obtained from Sigma-Aldrich, Life Technologies, and GE Healthcare Life Sciences, respectively. All solvents were purchased in septum-sealed bottles stored under an inert atmosphere. Reactions were monitored by LC/MS (Phenomenex Kinetex 2.1 mm × 30 mm 2.6 μm C18 100 Å column; 5 μL injection; 5–98% MeCN/H_2_O, linear gradient, with constant 0.1% v/v formic acid additive; 6 min run; 0.5 mL/min flow; ESI; positive ion mode). Reaction products were purified by preparative reverse phase HPLC (Phenomenex Gemini–NX 30 mm × 150 mm 5 μm C18 column). Analytical HPLC analysis was performed with an Agilent Eclipse XDB 4.6 mm × 150 mm 5 μm C18 column under the indicated conditions.

### JF_549_–CLIP-tag ligand

6-Carboxy-JF_549_ [12] (20 mg, 35.2 μmol) was combined with DSC (19.8 mg, 77.4 μmol, 2.2 eq) in DMF (1.5 mL). After adding Et3N (14.7 μL, 106 μmol, 3 eq) and DMAP (0.4 mg, 3.52 μmol, 0.1 eq), the reaction was stirred at room temperature for 2 h. Purification of the crude reaction mixture by reverse phase HPLC (10–95% MeCN/H2O, linear gradient, with constant 0.1% v/v TFA additive) afforded 18.3 mg (78%, TFA salt) of JF_549_-6-NHS as a dark purple solid. JF_549_-6-NHS (TFA salt, 5.3 mg, 8.0 μmol) and BC-NH_2_ (2.0 mg, 8.8 μmol, 1.1 eq) were combined in DMF (700 μL), and triethylamine (2.8 μL, 20.0 μmol, 2.5 eq) was added. The reaction was stirred at room temperature for 18 h while being shielded from light. It was subsequently purified by reverse phase HPLC (10–95% MeCN/H_2_O, linear gradient, with constant 0.1% v/v TFA additive) to provide 4.9 mg (79%, TFA salt) of the title compound as a dark red solid. Analytical HPLC: t_R_ = 9.9 min, 98.3% purity (10–95% MeCN/H_2_O, linear gradient, with constant 0.1% v/v TFA additive; 20 min run; 1 mL/min flow; ESI; positive ion mode; detection at 550 nm); MS (ESI) calculated for C_39_H_35_N_6_O_5_ [M+H]^+^ 667.3, found 666.9.

### ATTO 647N–HaloTag ligand

ATTO 647N NHS ester (5 mg, 5.9 μmol) and HaloTag amine (O2) ligand (2.0 mg, 8.9 μmol, 1.5 eq) were combined in DMF (1 mL), and DIEA (5.2 μL, 29.7 μmol, 5 eq) was added. The reaction was stirred at room temperature for 18 h while being shielded from light. It was subsequently purified by reverse phase HPLC (30–95% MeCN/H_2_O, linear gradient, with constant 0.1% v/v TFA additive) to provide 4.8 mg (84%, TFA salt) of the title compound as a dark blue gum. Analytical HPLC: t_R_ = 14.6/14.9 min (mixture of diastereomers), >99% purity (30–95% MeCN/H_2_O, linear gradient, with constant 0.1% v/v TFA additive; 20 min run; 1 mL/min flow; ESI; positive ion mode; detection at 650 nm); MS (ESI) calculated for C_52_H_72_ClN_4_O_4_ [M]^+^ 851.5, found 851.1.

### Alexa Fluor 594–HaloTag ligand

Alexa Fluor 594 NHS ester (5 mg, 6.1 μmol) and HaloTag amine (O2) ligand (2.0 mg, 9.2 μmol, 1.5 eq) were combined in DMF (1 mL), and DIEA (5.3 μL, 30.5 μmol, 5 eq) was added. The reaction was stirred at room temperature for 18 h while being shielded from light. It was subsequently purified by reverse phase HPLC (10–75% MeCN/H_2_O, linear gradient, with constant 0.1% v/v TFA additive) to provide 2.6 mg (41%, TFA salt) of the title compound as a violet solid. Analytical HPLC: t_R_ = 11.1 min, 98.7% purity (10–95% MeCN/H_2_O, linear gradient, with constant 0.1% v/v TFA additive; 20 min run; 1 mL/min flow; ESI; positive ion mode; detection at 600 nm); MS (ESI) calculated for C_45_H_55_ClN_3_O_12_S_2_ [M]^+^ 928.3, found 927.8.

### Cy2(Gly)–SNAP-tag ligand

Cy2 bis-NHS ester (5 mg, 5.8 μmol) was taken up in DMF (1 mL); DIEA (5.1 μL, 29.1 μmol, 5 eq) was added, followed by a solution of BG-NH_2_ (5 mg/mL in DMF, 160 μL, 3.0 μmol, 0.5 eq). After stirring for 1 h at room temperature, additional BG-NH_2_ (5 mg/mL in DMF, 123 μL, 2.3 μmol, 0.4 eq) was added. The reaction was stirred at room temperature for 1 h, concentrated *in vacuo*, and purified by reverse phase HPLC (10–50% MeCN/H_2_O, linear gradient, with constant 0.1% v/v TFA additive) to provide 2.5 mg of the Cy2(mono-NHS)-SNAP-tag ligand as an orange solid. This intermediate was combined with glycine (3 mg, 40.0 μmol, 20 eq) and DIEA (1.8 μL, 10.1 μmol, 5 eq) in DMF (1 mL). The reaction was stirred at room temperature for 18 h, concentrated *in vacuo*, and purified by reverse phase HPLC (10–40% MeCN/H_2_O, linear gradient, with constant 0.1% v/v TFA additive) to provide 0.5 mg (8%, TFA salt) of the title compound as an orange solid. Analytical HPLC: t_R_ = 8.6 min, >99% purity (10–50% MeCN/H_2_O, linear gradient, with constant 0.1% v/v TFA additive; 20 min run; 1 mL/min flow; ESI; positive ion mode; detection at 500 nm); MS (ESI) calculated for C_44_H_48_N_9_O_13_S_2_ [M]^+^ 974.3, found 974.1.

### Fly stocks

Flies were raised on standard corn meal molasses food. The stocks used in this paper included the following: *SS02565*: Stable split GAL4 stock 02565 consists of *55C09-p65ADZp in VK00027* and *VT040566-ZpGDBD in attP2* (*BJD_111C02_AV_01*; [14–16]); *SS00313*: Stable split GAL4 stock 00313 consists of *38C11-p65ADZp in attP40* and *59C10-ZpGdbd in attP2* [14,16]; *5XUAS-IVS-myr*::*smFLAG in VK00005*, *pJFRC51-3XUAS-IVS-Syt*::*smHA in su(Hw)attP1* [17]; *brp-SNAP* [10]; *UAS-myr*::*4xCLIPf in VK00005* [11]; *UAS-Syt*::*Halo7 in VK0027* [11]; *57C10-Flp2 in attp18;; pJFRC201-10XUAS>STOP>myr*::*smGFP-HA in VK00005*, *pJFRC240-10XUAS>STOP>myr*::*smGFP-V5-THS-10XUAS>STOP>myr*::*smGFP-FLAG in su(Hw)attP1* [13]; *20XUAS-Cs-Chrimson-mVenus trafficked in attP18* [17]; *UAS-7xHalo7*::*CAAX in VK00005* [11]; and *UAS-myr-Halo2 in attP2* [10].

### Dissection & fixation

For a detailed protocol, see S1 Protocol. One- to five-day old female flies were anesthetized with CO_2_, briefly submerged in cold 70% ethanol, briefly submerged in cold S2 medium (Schneider’s Insect Medium, S01416, Sigma Aldrich, St. Louis, MO), then held in additional cold S2 medium for up to 20 minutes until dissection. Brains and ventral nerve cords were dissected in cold S2 medium, then fixed in room temperature 2% paraformaldehyde in S2 medium for 55 minutes while rotating on a nutator. Samples were washed 1–4 times (fewer for chemical tags, four times for IHC) in phosphate-buffered saline plus 0.5% Triton X-100 (PBT) for 10–20 minutes each.

### Immunohistochemistry

IHC samples were held for up to two days at 4°C in PBT after dissection. Polarity labeling followed [17], with a detailed protocol in S2 Protocol. MCFO labeling followed [13], with a detailed protocol in S3 Protocol. Both protocols were modified by the replacement of Cy5 goat anti-rat or Alexa Fluor 647 goat anti-rat with ATTO 647N goat anti-rat (RRID: AB_10893386, polyclonal targeting Rat IgG (H&L) antibody, Rockland Immunochemicals Inc. 612-156-120, Limerick, PA) at their original concentrations.

### Chemical tagging

Samples were tagged following 1–4 post-fixation washes plus in some cases being held up to two hours in PBT, otherwise following the approach of [10]. Chemical tag ligands were applied in a 200 μL volume for 15 minutes at room temperature on a nutator, followed by two 10 minute washes. All ligands were used at 2 μM except CLIP-tag ligands, which were used at 3 μM. If both SNAP- and CLIP-tag ligands were used, the SNAP-tag ligands were applied first followed by CLIP-tag ligands, to minimize cross-reactivity. In addition to the novel chemical tag ligands described here, we used CLIP-Cell TMR-Star (S9219S, New England Biolabs, Ipswich, MA) and JF_549_ SNAP-tag ligand [12].

### Hybrid IHC & chemical tag

The hybrid IHC/chemical tag protocol combined Cy2 SNAP-tag ligand labeling of the brp-SNAP reference with antibody labeling of specific neurons. The procedure followed the full chemical tag procedure and then an IHC protocol modified to remove nc82 reference labeling (Polarity hybrid protocol https://dx.doi.org/10.17504/protocols.io.nycdfsw; MCFO hybrid protocol https://dx.doi.org/10.17504/protocols.io.nyhdft6). The processing required for IHC appeared to diminish the chemical tag signal somewhat when compared to pure chemical tagging.

### Dehydration, clearing & mounting

Most samples were fixed for four hours in room temperature 4% paraformaldehyde in PBS following labeling and before dehydration, in order to make the shrinkage during dehydration more uniform. They were then dehydrated in an ethanol series, cleared in xylene, and mounted in DPX, as described in [17], with a detailed protocol in S4 Protocol.

### Imaging

Samples were imaged on several Zeiss LSM 710 and 700 confocal scanning microscopes with either a Plan-Apochromat 20x/0.8 M27 or Plan-Apochromat 63x/1.40 oil immersion objective. All images are maximum intensity projections of captured confocal stacks. Imaging was performed using Zeiss ZEN software with a custom Multitime macro. Except as described below, the Multitime macro was allowed to automatically select appropriate laser power and gain for each sample. As a result, each image is independently scaled for intensity and was evaluated based on laser power and gain required in addition to image quality, with limited weight placed on raw intensity.

### Intensity quantification

After initial imaging, samples were re-imaged with fixed laser and gain settings for quantification of fluorescence intensity. Laser and detection settings were as follows:

Cy2: 488 nm laser, 498–543 nm detection range, 20% laser power, 560 gain

TMR & JF_549_: 561 nm laser, 561–620 nm detection range, 20% laser power, 340 gain.

Intensity was quantified for neuropil reference by opening 20X stacks in Fiji, moving in z to where the fan-shaped body comes together just beyond the ellipsoid body, drawing a 30-pixel diameter circle on the center of the fan-shaped body, and using Fiji’s histogram function (single slice) to measure the mean intensity inside the circle. Without changing depth, 30-pixel diameter mean intensities were measured from the brightest part of each medial optic lobe. The three measurements were averaged together for each sample. Intensity values were measured in arbitrary units of intensity between 0 and 4095.

Intensity values for *SS02565* neurons were measured similarly to the neuropil reference, but moving to the brightest z slice of expression in prominent projections to the anterior optic tubercle for each hemisphere. Mean intensity for a 30-pixel diameter region around each was measured and the two measurements were averaged together.

Samples that had been initially imaged with both 20X and 63X objectives had approximately 50% lower average intensity than those imaged only at 20X, presumably due to photo-bleaching, and were excluded from the reported averages. Raw intensity comparisons between fluorophores imaged with different lasers and detectors, e.g. Cy2 SNAP-tag ligand and JF_549_ SNAP-tag ligand, unfortunately could not be meaningfully quantitatively performed.

## Results and discussion

**Fig 2:**
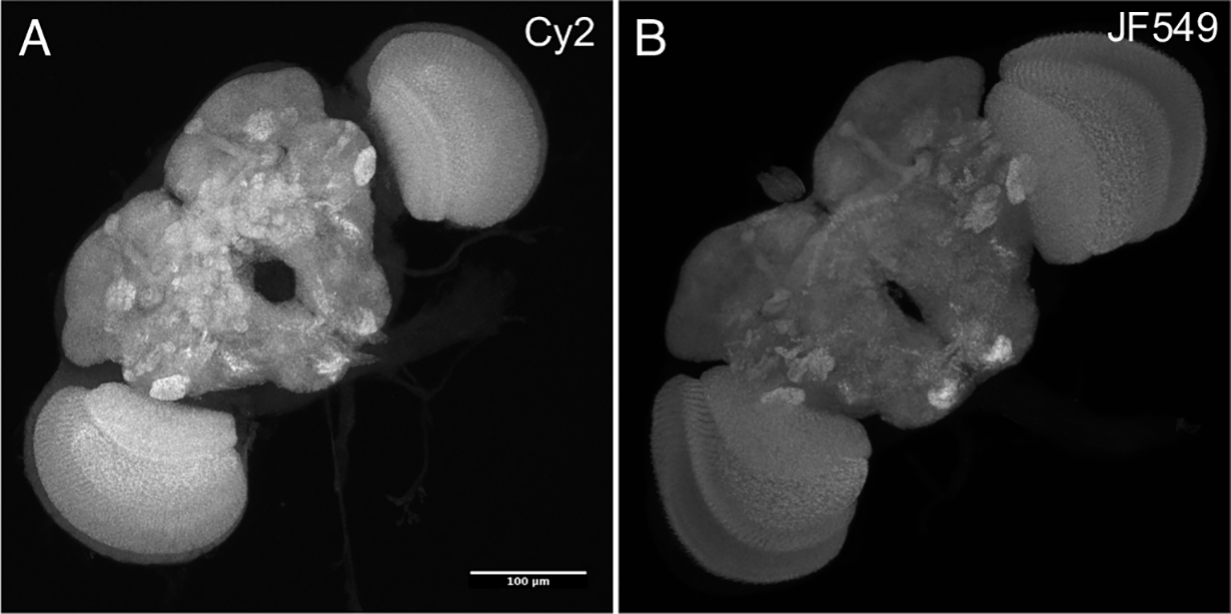
Comparison of *brp-SNAP* with JF_549_ or Cy2 SNAP-tag ligands. *brp-SNAP* flies had brains removed, fixed, and incubated for 15 minutes with (A) Cy2 SNAP-tag ligand, or (B) JF_549_ SNAP-tag ligand. Cy2 samples had additional 4 hour 4% post-fixation, improving their morphology during dehydration and DPX mounting.

We first investigated chemical tagging to label the Drosophila neuropil as a reference for nervous system morphology. We evaluated two different dye ligands for the Brp-SNAP-tag neuropil marker: a novel 488 nm-excited Cy2 SNAP-tag and the known 560 nm-excited JF_549_ SNAP-tag ligands [10,11]. Although not as bright as anti-Brp nc82 antibody and Cy2 anti-mouse secondary (422.9 ± 76.6 arbitrary intensity units (a.u.), standard deviation, n = 12), the chemical tagging protocol was substantially faster and both dyes showed bright specific staining of fly neuropil in DPX (Cy2: 188.6 ± 27.2 a.u., standard deviation, n = 21; **Fig 2**). The higher brightness of the IHC protocol likely stems from the inherent amplification at both the tag and primary-to-secondary antibody stages (see methods; [11,18]). We selected Cy2 SNAP-tag ligand for further work due to its direct replacement of the Cy2 antibody in current use (https://www.janelia.org/project-team/flylight/protocols).

**Fig 3:**
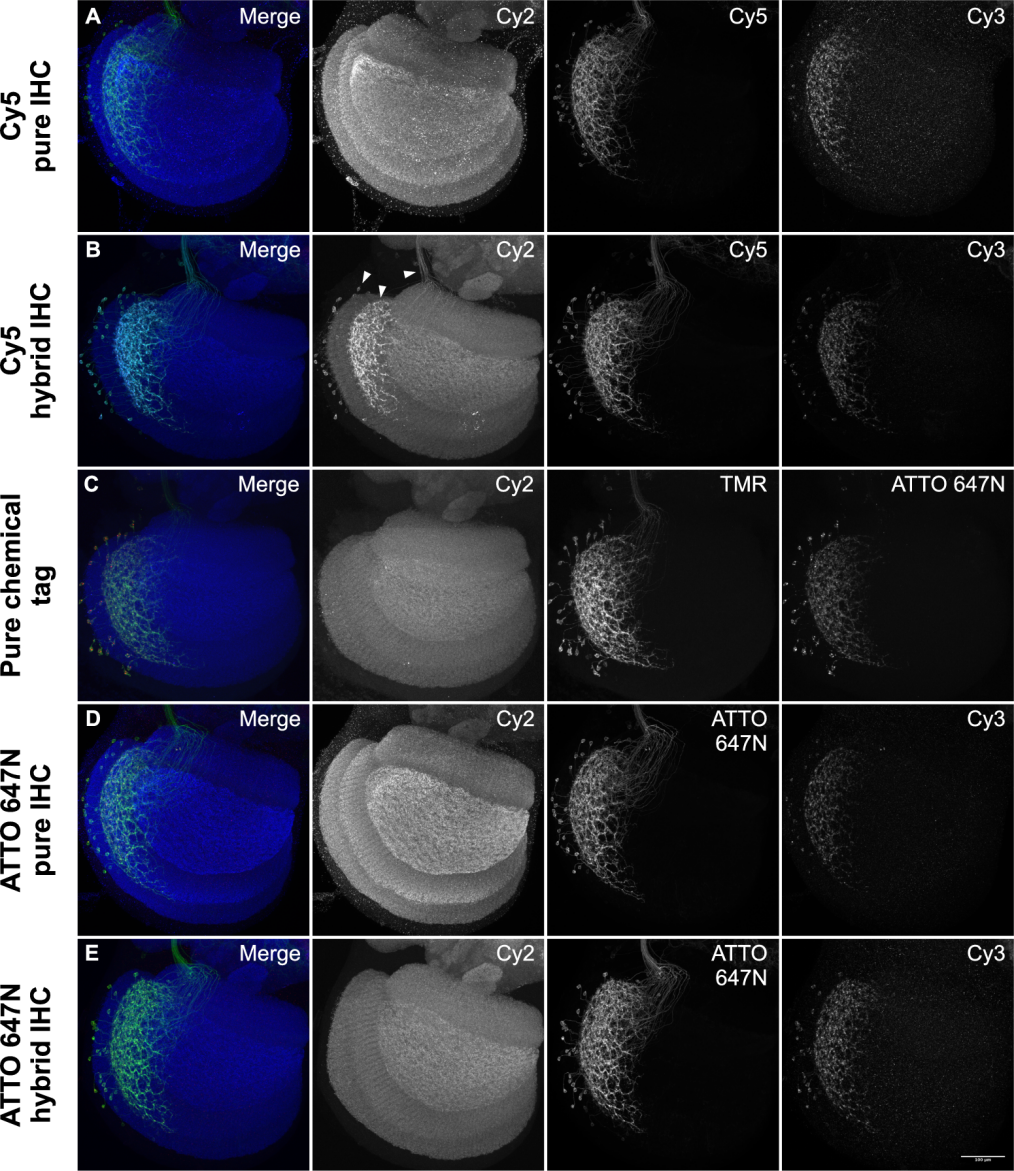
Comparison of Polarity IHC and chemical tag labeling methods. All samples show the Drosophila left optic lobe imaged at 63X. Each image is independently scaled for optimal intensity. (A). **Polarity pure IHC**: Split GAL4 *SS02565* was crossed to *w;; 5XUAS-IVS-myr*::*smFLAG in VK00005*, *pJFRC51-3XUAS-IVS-Syt*::*smHA in su(Hw)attP1* and was labeled over a period of 13 days with nc82 mouse anti-Brp/Cy2 anti-mouse, rabbit anti-HA/Cy3 anti-rabbit, and rat anti-FLAG/Cy5 anti-rat. (B). **Polarity hybrid IHC**: *SS02565* was crossed to *w; brp-SNAP; 5XUAS-IVS-myr*::*smFLAG in VK00005*, *pJFRC51-3XUAS-IVS-Syt*::*smHA in su(Hw)attP1* and labeled with Cy2 SNAP-tag ligand for 15 minutes, followed by rabbit anti-HA/Cy3 anti-rabbit, and rat anti-FLAG/Cy5 anti-rat over 6 days. Arrowheads indicate bleed-through of Cy5 into Cy2 channel. (C). **Polarity pure chemical tag**: *SS02565* was crossed to *w; brp-SNAP; UAS-myr*::*4xCLIPf in VK00005*, *UAS-Syt*::*Halo7 in VK0027* and labeled for 15 minutes with Cy2 SNAP-tag ligand, TMR CLIP-tag ligand, and ATTO 647N HaloTag ligand. (D). **Polarity ATTO 647N pure IHC**: As in (A) but with ATTO 647N instead of Cy5. (E). **Polarity ATTO 647N hybrid IHC**: As in (B) but with ATTO 647N instead of Cy5.

**Fig 4:**
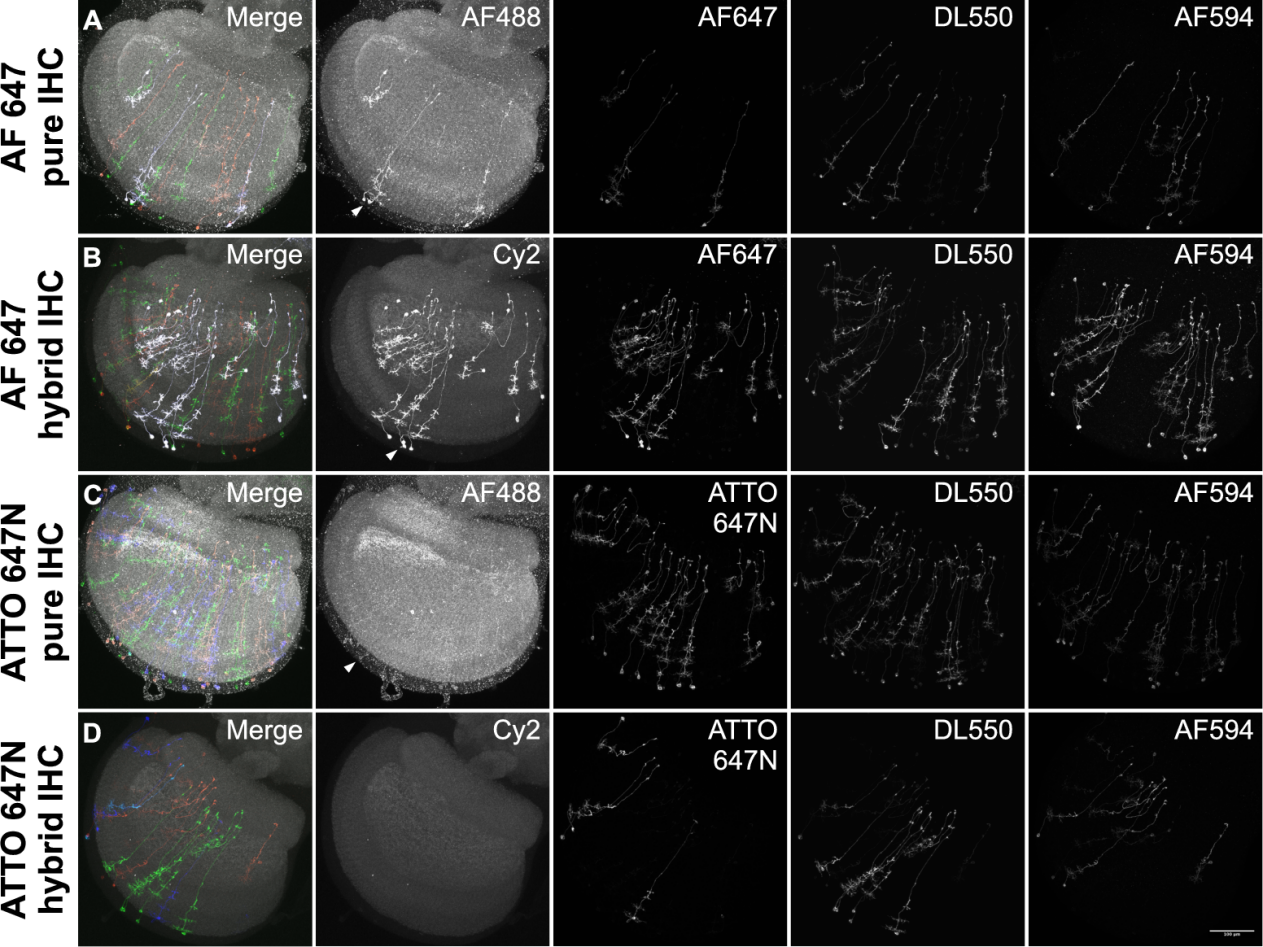
Comparison of MCFO IHC and chemical tag labeling methods. All samples show the Drosophila left optic lobe imaged at 63X. Each image is independently scaled for optimal intensity. Arrowheads indicate bleed-through into Cy2/AF488 channel. (A). **MCFO pure IHC**: Split GAL4 *SS00313* was crossed to *57C10-Flp2 in attp18;; pJFRC201-10XUAS>STOP>myr*::*smGFP-HA in VK00005*, *pJFRC240-10XUAS>STOP>myr*::*smGFP-V5-THS-10XUAS>STOP>myr*::*smGFP-FLAG in su(Hw)attP1*, and was labeled with nc82 mouse anti-Brp/Alexa Fluor 488 anti-mouse, rat anti-FLAG/Alexa Fluor 647 anti-rat, rabbit anti-HA/Alexa Fluor 594 anti-rabbit, and DyLight 550 mouse anti-V5 over a period of 7 days. (B). **MCFO hybrid IHC**: *SS00313* was crossed to *57C10-Flp2 in attp18; brp-SNAP; pJFRC201-10XUAS>STOP>myr*::*smGFP-HA in VK00005*, *pJFRC240-10XUAS>STOP>myr*::*smGFP-V5-THS-10XUAS>STOP>myr*::*smGFP-FLAG in su(Hw)attP1* and labeled for 15 minutes with Cy2 SNAP-tag ligand, followed by rat anti-FLAG/Alexa Fluor 647 anti-rat, rabbit anti-HA/Alexa Fluor 594 anti-rabbit, and DyLight 550 mouse anti-V5 over a period of 6 days. (C). **MCFO ATTO 647N pure IHC**: As in (A) but with ATTO 647N instead of Alexa Fluor 647. (D). **MCFO ATTO 647N hybrid IHC**: As in (B) but with ATTO 647N instead of Alexa Fluor 647.

We tested the new Cy2 SNAP-tag ligand in combination with previously described chemical tag and antibody reagents to determine the optimal approach for two labeling schemes: (1) ‘Polarity’, to label neuronal membrane and presynaptic sites (**Fig 3**); and (2) ‘MultiColor FlpOut’ (MCFO), to characterize the morphology of individual neurons by stochastic labeling ([13]; **Fig 4**). The current Polarity scheme consists of neuropil reference, a myristoylated reporter for neuronal membrane, and a Synaptotagmin-fused reporter for presynaptic terminals: *5XUAS-IVS-myr*::*smFLAG in VK00005*, *pJFRC51-3XUAS-IVS-Syt*::*smHA in su(Hw)attP1* [17]. The MCFO scheme consists of neuropil reference plus three stochastically activated membrane reporters: *pJFRC201-10XUAS>STOP>myr*::*smGFP-HA in VK00005*, *pJFRC240-10XUAS>STOP>myr*::*smGFP-V5-THS-10XUAS>STOP>myr*::*smGFP-FLAG in su(Hw)attP1* [13].

For the Polarity protocol, we compared three different labeling strategies: (1) the existing pure IHC approach (**Fig 3A**); (2) a hybrid approach where the neuropil reference was labeled with *brp-SNAP*/Cy2-SNAP-tag ligand and specific neurons labeled with antibodies (**Fig 3B**); and (3) a pure chemical tag approach using *brp-SNAP*, *UAS-myr*::*4xCLIPf in VK00005*, *and UAS-Syt*::*Halo7 in VK0027* transgenes (**Fig 3C**). As in the case of neuropil labeling alone, the Polarity protocol signal quality of the chemical tagging systems was worse than for pure IHC. Moreover, in the hybrid protocol, the Cy2 chemical tag reference labeling exhibited an average intensity of 114.0 ± 20.0 a.u., standard deviation, n = 11, suggesting that the requisite processing steps for IHC lowered the chemical tag signal quality (see below). The results for the MCFO hybrid protocol were similar, with lower signal levels than the pure IHC MCFO protocol (**Fig 4A and 4B**).

In the pure IHC Polarity & MCFO protocols, and especially in the hybrid approaches, we observed signal from Cy5 (Polarity) or Alexa Fluor 647 (MCFO) in the 488 reference channel (**Fig 3A and 3B and Fig 4A and 4B**). This ‘bleed-through’ may be due to reported impurities in these cyanine-based dyes and/or due to fluorescence changes when embedded in DPX [19]. When we replaced both dyes with the rhodamine-based ATTO 647N we saw an elimination of bleed-through with the hybrid protocol and a strong reduction in bleed-through with pure IHC (**Fig 3D and 3E, Fig 4C and 4D**).

**Fig 5:**
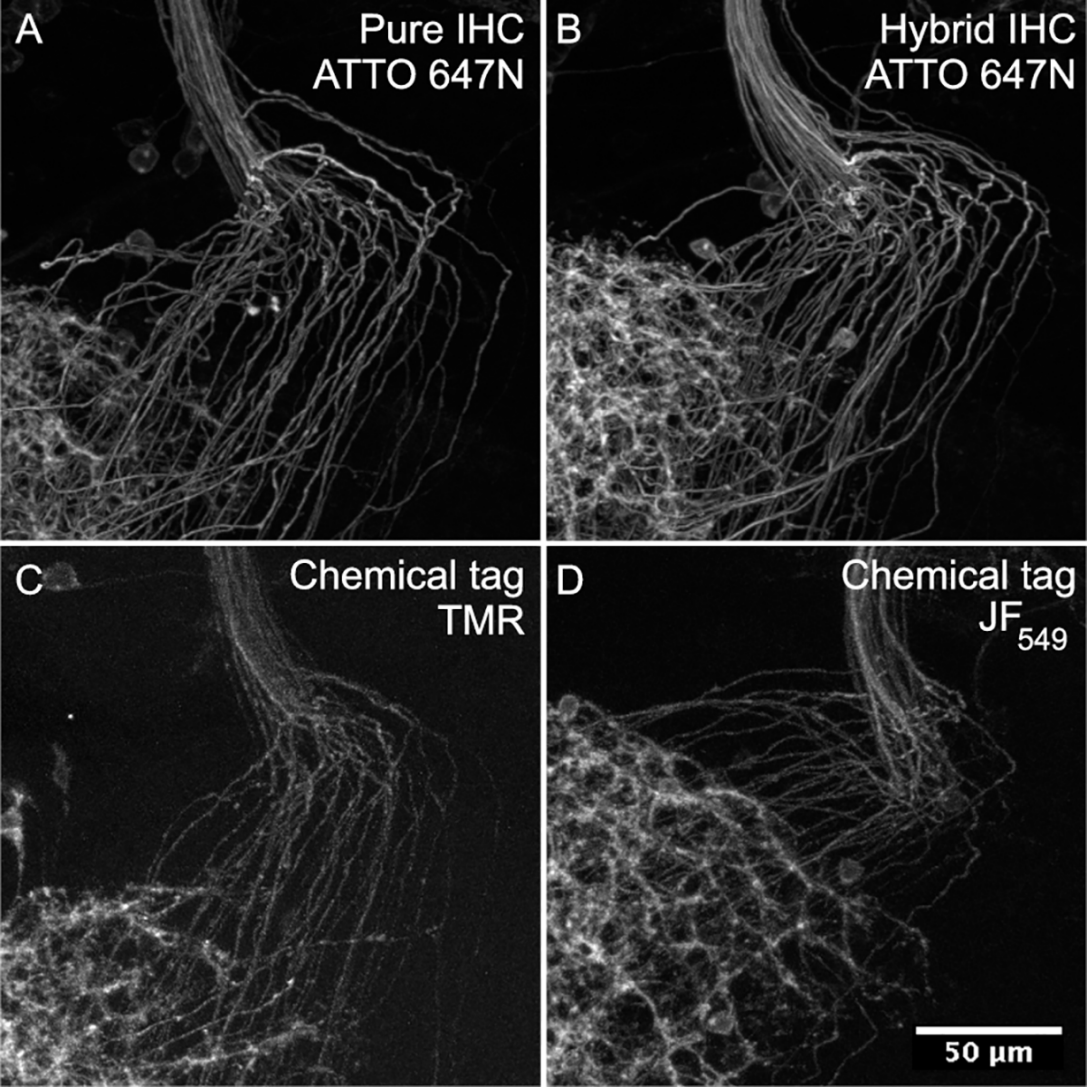
Detail comparison of *SS02565* neuronal membrane labeling. All samples show the same region of projections crossing from the left optic lobe to the central brain. Only the neuronal membrane channel is shown, and is labeled via antibodies in (A-B) and CLIP-tag in (C-D). (A) *SS02565* was crossed to *w;; 5XUAS-IVS-myr*::*smFLAG in VK00005*, *pJFRC51-3XUAS-IVS-Syt*::*smHA in su(Hw)attP1* and brains were labeled with pure IHC, including rat anti-FLAG and ATTO 647N goat anti-rat antibodies over a period of 13 days. (B) *SS02565* was crossed to *w; brp-SNAP; 5XUAS-IVS-myr*::*smFLAG in VK00005*, *pJFRC51-3XUAS-IVS-Syt*::*smHA in su(Hw)attP1* and brains were labeled with hybrid IHC, including rat anti-FLAG and ATTO 647N goat anti-rat antibodies over a period of 6 days. (C) *SS02565* was crossed to *w; brp-SNAP; UAS-myr*::*4xCLIPf in VK00005*, *UAS-Syt*::*Halo7 in VK0027* and brains were labeled for 15 minutes with pure chemical tags, including TMR CLIP-tag ligand. (D) *SS02565* was crossed to *w; brp-SNAP; UAS-myr*::*4xCLIPf in VK00005*, *UAS-Syt*::*Halo7 in VK0027* and brains were labeled for 15 minutes with pure chemical tags, including JF_549_ CLIP-tag ligand.

**Fig 6:**
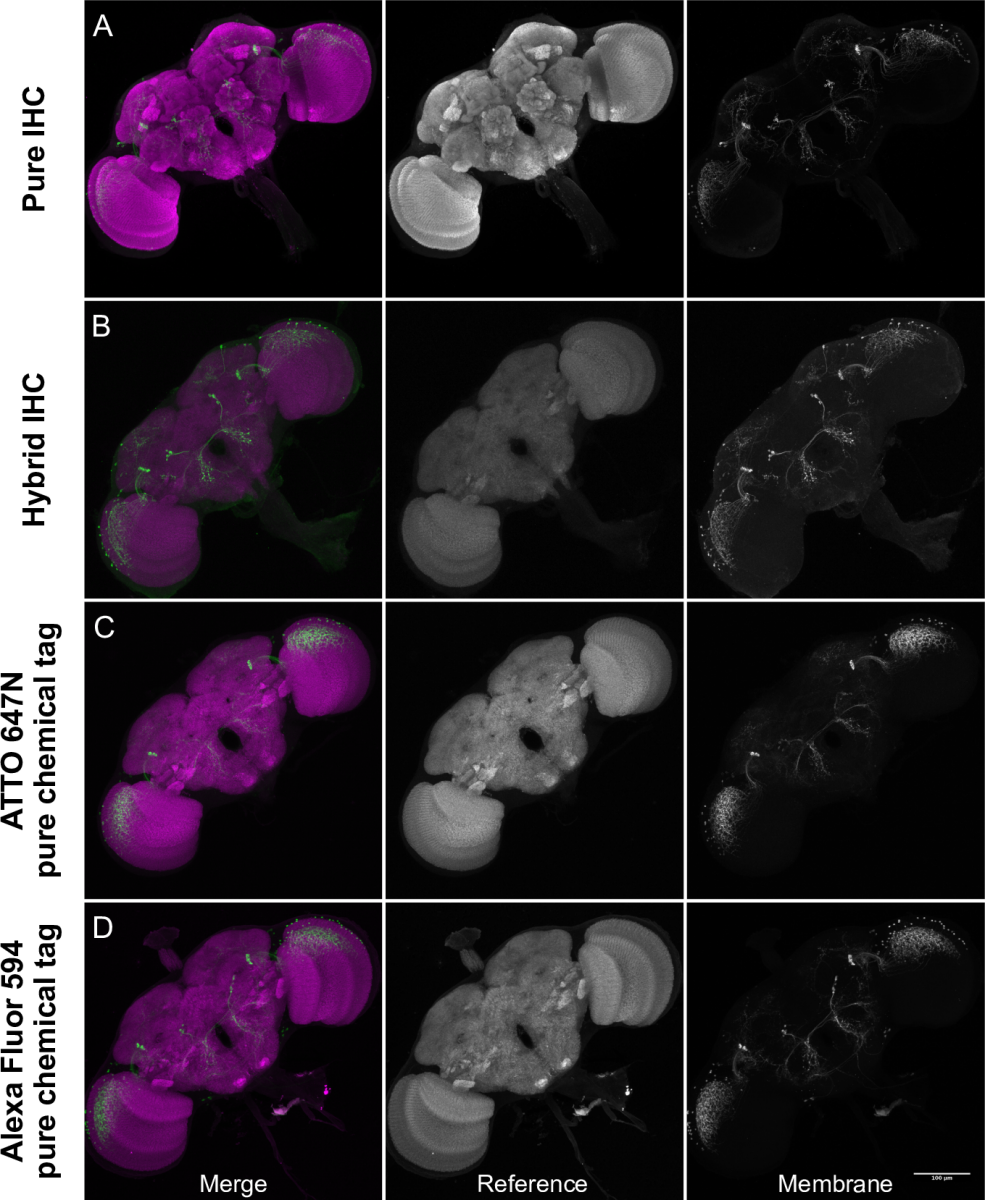
Comparison of reference and *SS02565* neuronal membrane labeling. (A) *SS02565* was crossed to *20XUAS-Cs-Chrimson-mVenus trafficked in attP18* and brains were labeled with pure IHC over a period of 7 days.(B) *SS02565* was crossed to *20XUAS-Cs-Chrimson-mVenus trafficked in attP18; brp-SNAP* and brains were labeled with hybrid IHC over a period of 7 days.(C) *SS02565* was crossed to *brp-SNAP; UAS-7xHalo7*::*CAAX in VK0005* and brains were labeled for 15 minutes with pure chemical tags, including ATTO 647N HaloTag ligand. (D) *SS02565* was crossed to *brp-SNAP; UAS-myr-Halo2 in attP2* and brains were labeled for 15 minutes with pure chemical tags, including Alexa Fluor 594 HaloTag ligand.

Closer examination of the pure chemical tag Polarity protocol showed that membrane-labeling was suboptimal at high resolution (**Fig 5**). To enhance the signal, we replaced tetramethylrhodamine (TMR) CLIP-tag ligand (33.1 ± 7.1 a.u., standard deviation, n = 11) with newly synthesized JF_549_ CLIP-tag ligand (60.2 ± 2.4 a.u., standard deviation, n = 5), which improved signal substantially, but still was not as bright as IHC. We also compared the effectiveness of Alexa Fluor 594 HaloTag, and ATTO 647N HaloTag ligands in a two-color reference plus membrane label protocol (**Fig 6**). Both performed similarly well when paired with brp-SNAP and Cy2 SNAP-tag ligand.

In general, we observed that the processing speed improvements of chemical tag labeling are substantial—the time required to label tissue is greatly reduced from 1–2 weeks for IHC to an hour for chemical tags. However, despite recent improvements in transgenes and the new ligands reported here, the signal strength for chemical tagging was still lower than for the optimized IHC protocol. We expect both strategies to be useful depending on the experimental needs: while screening efforts benefit from the fast turnaround of chemical tagging, more detailed anatomical mapping efforts require the higher signal of IHC. In addition, the chemical tags method enables an alternative approach in cases of IHC antibody cross-reactivity. Finally, these data highlight the value of continuing development of new, brighter fluorophores for use with emerging labeling strategies in tissue.

## Supporting information

S1 Protocol Dissection and 2% fixation for Adult CNS. (PDF)

S2 Protocol Polarity sequential IHC for Adult CNS. (PDF)

S3 Protocol MCFO IHC for Adult CNS. (PDF)

S4 Protocol DPX mounting of Adult CNS. (PDF)
