# Supplementary material for: Optimization of fluorophores for chemical tagging and immunohistochemistry of Drosophila neurons
Source: PLoS One. 2018 Aug 15;13(8):e0200759. doi: 10.1371/journal.pone.0200759 (PMC6093644; doi:10.1371/journal.pone.0200759)
Supplement: S1 Protocol — (PDF) [file pone.0200759.s001.pdf]

SUMMARY –PROTOCOL – DISSECTION AND 2% PFA FIXATION FOR ADULT DROSOPHILA CNS

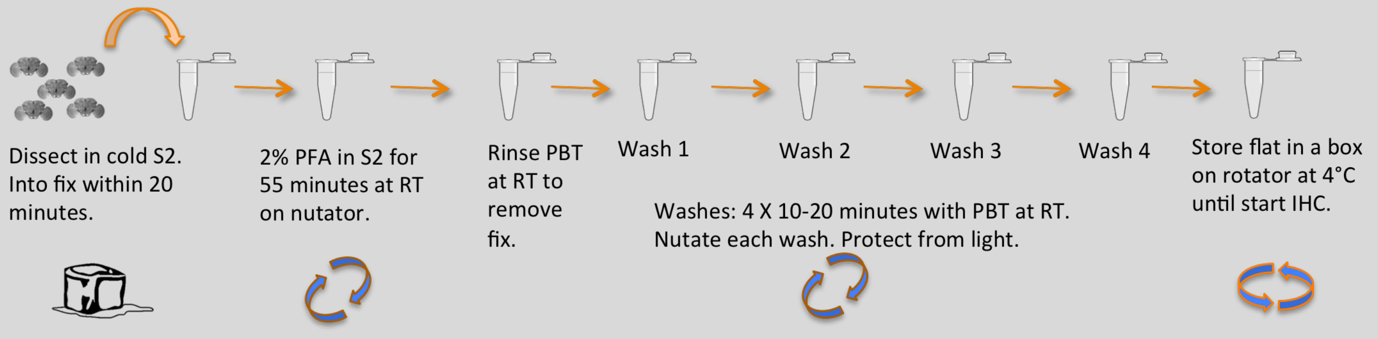

Protocol based on collaboration with R.M. Johnston and based on work described in:

Nern A, Pfeiffer BD, Rubin GM. Optimized tools for multicolor stochastic labeling reveal diverse stereotyped cell arrangements in the fly visual system. Proc Natl Acad Sci U S A. 2015; 112: E2967-76. doi: 10.1073/pnas.1506763112

Aso Y, Hattori D, Yu Y, Johnston RM, Iyer NA, Ngo T-TB, et al. The neuronal architecture of the mushroom body provides a logic for associative learning. Elife. 2014; 3: e04577. doi: 10.7554/eLife.04577

Dissection

1. **De-wax Anesthetized Flies**.

   - Prepare three wells in a glass well plate: one with 70% ethanol and the other two with cold Schneider's Insect Medium (S2). Place a sticky dot next to the 70% well to clearly distinguish it from the other wells filled with S2.
   - Anesthetize flies with CO_2 or cold. Grasp a fly by the wings or legs with forceps and briefly submerge the anesthetized fly first in 70% ethanol (2 seconds) followed immediately by a brief dip in the first well of cold S2. Then submerge the fly in the second well of cold S2 where it will remain until dissected.
   - Do not use a transfer pipette to move flies between wells because this adds ethanol to the S2 wells. Extended exposure to ethanol will accelerate denaturation of the tissue and fluorescent proteins making the tissue unusable.
   - Keep the dish with flies submerged in S2 on ice until they are dissected.
   - *Do not anesthetize and rinse more flies than can be dissected within **20 minutes***. Flies kept submerged longer will die and their brains will be unusable due to postmortem changes

2. **Dissect** in cold S2. Transfer the rinsed fly to a Sylgard-lined dish with cold S2 and dissect.

   - Replace your puddle of S2 with fresh COLD S2 when it becomes littered with dissection debris.
   - Dissect only as many flies as you can comfortably complete in **20 minutes** before transferring the dissected brains to fixative. All fixations are precisely timed (see next).

Fixation – 2% PFA in S2 for 55-60 minutes

3. **Timed Fixation**. Within 20 minutes of dissection, transfer tissue to a 2mL Protein LoBind tube with ~1.9 mL of 2% paraformaldehyde (PFA) in S2 at room temperature (RT) and incubate for 55-60 minutes at RT while on a nutator. Cover tissue to protect from light.
    - *OPTIMAL* fixation time is **55-60 minutes for 2% PFA in S2**. Use a timer to precisely time the duration of your fixation.
4. **Fix Removal - Rinse**. Place the tubes upright to allow the tissue to sink. Use a transfer pipetted to aspirate the fix and fill tube with phosphate buffered saline with 5% Triton X-100 (PBT) at RT. Invert the tube a few times. Let the tissue settle and aspirate the rinse solution with a transfer pipette.
    - To help the tissue sink, add a small amount (~15 µL) of PBT at RT to the tube. The detergent (Triton) in the PBT displaces air trapped in the trachea, helping the tissue sink.
5. **Fix removal - Washes**. Add 1.75 mL of RT PBT and nutate for 10-20 minutes at RT. Repeat for a total of 4 washes. Protect samples from light during washes.
6. **Storage.** Store the tubes of tissue in 0.5% PBT at 4°C. Nutate or lay the tubes flat in a covered box on a rotator. Do not store upright. Protect from light.
    - Typically, these tubes will begin the IHC (immunohistochemistry) process the following day but can be stored for up to 3 days. If stored more than overnight, aspirate the old PBT and do a brief wash with 0.5% PBT before beginning IHC processing.
