# Supplementary material for: Optimization of fluorophores for chemical tagging and immunohistochemistry of Drosophila neurons
Source: PLoS One. 2018 Aug 15;13(8):e0200759. doi: 10.1371/journal.pone.0200759 (PMC6093644; doi:10.1371/journal.pone.0200759)
Supplement: S3 Protocol — (PDF) [file pone.0200759.s003.pdf]

MCFO IHC for Adult CNS

- All tissues and solutions are at room temperature (RT), unless noted. Always protect tissue from light exposure.
- For details on dissection and fixation see Protocol - Adult Dissection and 2% Fixation.
- For mounting and embedding instructions refer to Protocol – DPX Mounting.

Protocol based on collaboration with R.M. Johnston and based on work described in:

Nern A, Pfeiffer BD, Rubin GM. Optimized tools for multicolor stochastic labeling reveal diverse stereotyped cell arrangements in the fly visual system. Proc Natl Acad Sci U S A. 2015; 112: E2967-76. doi: 10.1073/pnas.1506763112

1. **Dissect**. Dissect adult brains or CNS in cold Schneider's Insect Medium (S2).
2. **Fix**. Transfer tissue to 2 mL Protein LoBind tubes filled with 2% paraformaldehyde (PFA) in S2 at RT. Fix for 55-60 minutes at RT while nutating.
3. **Post-fix wash**. Remove the fix and add 1.75 mL phosphate buffered saline with 0.5% Triton X-100 (PBT) and wash for a total of 4 X 10-minutes washes while nutating. If needed, store tissue in 0.5% PBT at 4°C while nutating or lay tube flat and rotate.
4. **Block Goat Serum (GS)**. Remove PBT and add 200 µL 5% GS in PBT per tube. Incubate for 1.5 hours at RT on a rotator with tubes upright.
5. **Primary antibodies**. Remove block and add primary antibodies diluted in 5% GS in PBT for a volume of 200 µL per tube. Incubate for 4 hours at RT on a rotator with tubes upright. Then continue incubation at 4°C on a rotator with tubes upright for 2 overnights.
    - Mouse nc82 (1:30 or 33.3 µL/mL)
    - Rat α-FLAG Tag (1:200 or 5 µL/mL)
    - Rabbit α-HA Tag (1:300 or 3.3 µL/mL)
6. **Post-primary washes.** Remove the primary antibody and do a brief rinse with 1.75 mL 0.5% PBT. Allow the tissue to settle to the bottom and then remove the rinse solution and add 1.75 mL 0.5% PBT. Wash for a total of 5 X 15-minute washes while nutating.
7. **Secondary antibodies**. Remove PBT and add the secondary antibodies diluted in 5% GS in PBT for a volume of 200 µL per tube. Incubate for 4 hours at RT on a rotator with tubes upright. Then continue incubation at 4°C on a rotator with tubes upright for 3-4 overnights.
    - Cy2 Goat α-Mouse (1:600 or 1.67 µL/mL)
    - ATTO647N Goat α-Rat (1:300 or 3.3 µL/mL)
    - AF594 Donkey α-Rabbit (1:500 or 2 µL/mL)
8. **Post-secondary washes.** Remove the secondary antibody and do a brief rinse with 1.75 mL 0.5% PBT. Allow the tissue to settle to the bottom and then remove the rinse solution and add 1.75 mL 0.5% PBT. Wash for a total of 5 X 15-minute washes while nutating. If needed, store tissue in 0.5% PBT at 4°C while nutating or lay tube flat and rotate.
9. **Block Normal Mouse Serum (NMS)**. Remove PBT and add 200 µL of 5% NMS in PBT per tube. Incubate for 1.5 hours at RT on a rotator with tubes upright

10. **Direct Label α-V5 antibody**. Remove NMS block and add DL550 Mouse α-V5 in 5% NMS in PBT. Incubate for 4 hours at RT on a rotator with tubes upright. Then continue incubation at 4°C on a rotator with tubes upright for 1 overnight.
    - DL550 Mouse α-V5 (1:500 or 2 µL/mL)
11. **Post- α-V5 washes.** Remove the α-V5 antibody and do a brief rinse with 1.75 mL 0.5% PBT. Allow the tissue to settle to the bottom and then remove the rinse solution and add 1.75 mL 0.5% PBT. Wash for a total of 5 X 15-minute washes while nutating.
12. **Pre-embedding fixation**. Remove PBT and add 1.75 mL 4% PFA in PBS at RT. Fix for 4 hours at RT while nutating.
13. **Post-4% PFA washes.** Remove the 4% PFA and do a brief rinse with 1.75 mL 0.5% PBT. Allow the tissue to settle to the bottom and then remove the rinse solution and add 1.75 mL 0.5% PBT. Wash for a total of 4 X 15-minute washes while nutating. If needed, store tissue in 0.5% PBT at 4°C while nutating or lay tube flat and rotate.
14. **Mount**. Mount the tissue on a poly-L-lysine (PLL) coated cover glass.
15. **Dehydrate**. Move the cover glass through a series of 7 cover glass staining jars filled with increasing concentrations of ethanol (30%, 50%, 75%, 95%, 100%, 100%, 100%). Soak the cover glass for 10 minutes in each jar.
16. **Xylene clearing**. (IN THE HOOD). Move the cover glass through a series of 3 jars filled with xylene. Soak the cover glass for 5 minutes in each jar.
17. **DPX embedding**. Add 7 drops of dibutyl phthalate in xylene (DPX) on top of the tissue mounted on the cover glass. Place the cover glass (DPX down) on a prepared slide with spacers. Use the edge of a glass slide to gently press down on the center of the cover glass to seat the cover glass onto the slide. Let the slide dry in the hood for 2 days before viewing.

Reporter Genotype

- pBPhsFlp2::PEST in attP3; ;pJFRC201-10XUAS-FRT>STOP>FRT-myr::smGFP-HA in VK0005, pJFRC240-10XUAS-FRT>STOP>FRT-myr::smGFP-V5-THS-10XUAS-FRT>STOP>FRT-myr::smGFP-FLAG in su(Hw)attP1

Reagents and Supplies

- Cy2 Goat α-Mouse. Jackson Immuno Research. # 115-225-166
- AF594 Donkey α-Rabbit. Jackson Immuno Research. # 711-585-152
- ATTO 647N Goat α-Rat IgG (H&L) Antibody. Rockland. # 612-156-120
- DL550 Mouse α-V5 Tag. AbD Serotec. # MCA1360D550GA
- DPX Mountant for Microscopy. Electron Microscopy Sciences. # 13512, 500 mL
- Ethanol, ACS reagent, >99.5% (200 proof). Sigma Aldrich. # 459844-1L
- GS – Goat Serum. Life Technologies. 16210-064, 100 mL
- Kodak Photo-Flo 200 Solution. Electron Microscopy Sciences. # 74257
- nc82 – Mouse α-bruchpilot. Developmental Studies Hybridoma Bank. # nc82-s
- NMS – Normal Mouse Serum. Jackson Immuno Research. # 015-000-120
- PBS – Phosphate Buffered Saline, 1X. Cellgro. # 21-040
- PFA – Paraformaldehyde. 20% PFA. Electron Microscopy Sciences. # 15713-S
- Poly-L-Lysine. Sigma Aldrich. # P1524-25MG
- Protein LoBind Microcentrifuge Tubes - 2 mL. Eppendorf. # 022431102
- S2 – Schneider's Insect Medium. Sigma Aldrich. # S01416
- Rabbit α-HA Tag. Cell Signal Technologies. # 3724S
- Rat α-FLAG Tag (DYKDDDDK Epitope Tag). Novus Biologicals. # NBP1-06712
- Triton X-100. Sigma Aldrich. # X100
- Xylenes. Fisher Scientific. # X5-500
