# Supplementary material for: Optimization of fluorophores for chemical tagging and immunohistochemistry of Drosophila neurons
Source: PLoS One. 2018 Aug 15;13(8):e0200759. doi: 10.1371/journal.pone.0200759 (PMC6093644; doi:10.1371/journal.pone.0200759)
Supplement: S4 Protocol — (PDF) [file pone.0200759.s004.pdf]

DPX Mounting of *Drosophila* CNS

SUMMARY

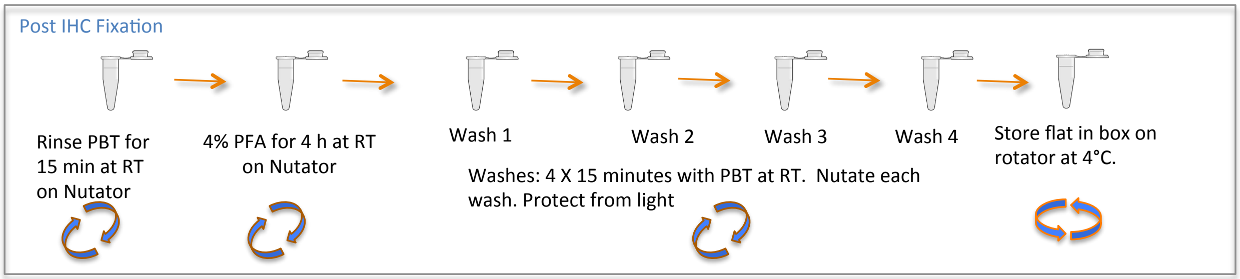

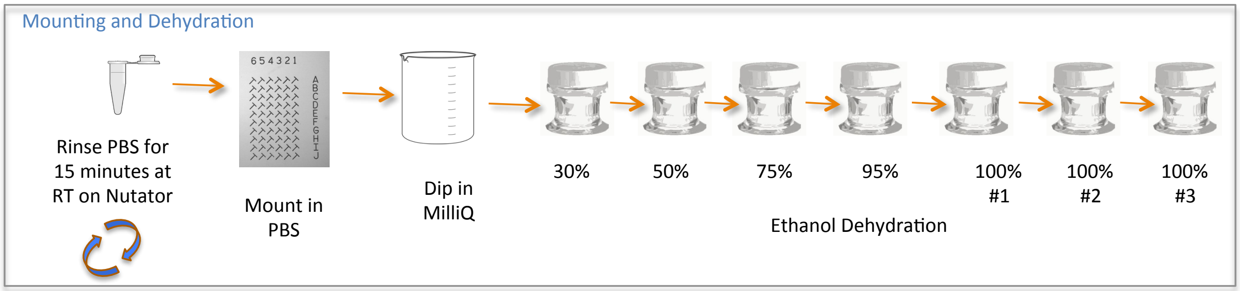

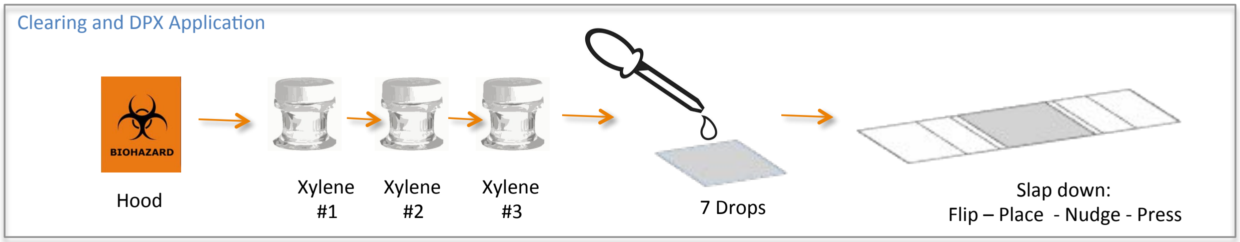

1. **Prep – day before**
   - Dip cover glass in PLL and make slides
2. **Secondary Fix – day before**
   - Fix in 4% PFA in PBS for 4 hours while nutating at RT. 4 X 15-minute washes in PBT.
3. **Mount**
   - Rinse tissue in PBS for 10 minutes while nutating to remove PBT.
   - Place tissue on the PLL-dipped cover glass using PBS to keep tissue hydrated.
   - Prior to dehydration, dip the cover glass with mounted tissues in MilliQ for 1-2 seconds to remove salts.
4. **Dehydration Series**
   - 10 minutes soaks in successive baths of ethanol: 30%, 50%, 75%, 95%, 100%, 100%, 100%.
5. **Xylene Clearing** (in the hood)
   - Soak for 5 minutes each in three successive baths of xylene (3 X 5-minutes).
6. **DPX application** (in the hood)
   - Add 7 drops of DPX to the tissue on the cover glass. Wait for it to spread out evenly to the edges (~30 s).
7. **Position the cover glass: "Flip – Place – Nudge – Press"** (in the hood)
   - Flip the cover glass over and place it down on the prepared slide between the spacers.
   - Use the tip of a glass slide to center the cover glass between the spacers. *Gently* press down once in the middle of the cover glass using the tip of one edge of a glass slide.
8. **Drying**
   - Let the slide dry in the hood for 24 hours before handling. Cure for 2-3 days at RT before imaging.

Protocol based on collaboration with R.M. Johnston and based on work described in:

Nern A, Pfeiffer BD, Rubin GM. Optimized tools for multicolor stochastic labeling reveal diverse stereotyped cell arrangements in the fly visual system. Proc Natl Acad Sci U S A. 2015; 112: E2967-76. doi: 10.1073/pnas.1506763112

Aso Y, Hattori D, Yu Y, Johnston RM, Iyer NA, Ngo T-TB, et al. The neuronal architecture of the mushroom body provides a logic for associative learning. Elife. 2014; 3: e04577. doi: 10.7554/eLife.04577

DPX MOUNTING OF *DROSOPHILA* CNS

Material Preparation - day before

1. Prepare several cover glass (22x22 mm, no. 1) dipped in poly-L-lysine (PLL) in advance.
   - The day before mounting - one in-and-out dip in cold PLL. Let dry overnight in a drying rack at room temperature (RT) protected from dust and light (Figure S4A).
   - The number of dips can be adjusted to suit the "stickiness" preference of the mounter. If multiple dips are preferred it is advised to allow at least one hour of drying between successive dips.
   - Touch the dripping edge of the dipped cover glass to the lip of the PLL jar to remove any excess before placing the cover glass in the drying rack. Do not touch the cover glass to a Kimwipe to remove the drip of PLL. Kimwipe particles transfer easily to the cover glass and possibly back to the PLL jar on subsequent dips. Kimwipe particles fluoresce and interfere with imaging.

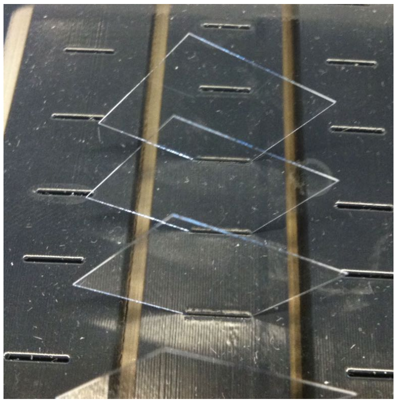

Figure S4A. Cover glass in a drying rack.

2. Prepare several slides in advance using the slide-making jig.
   - Score a 22x22 mm, no. 2 cover glass down the midline with a diamond cleaver using a glass slide as a straight edge. Snap the cover glass into two halves. Each half will act as a spacer between the cover glass with the mounted tissue and the surface of the glass slide.
   - Using the jig that defines a 1.2 cm region in the center of the slide (Figure S4B), align the cut spacers in the cut-outs so that the factory cut edges face toward the center of the 1.2 cm landing strip. The snapped or cut edges should face away from the center.
   - Secure the spacers with tape using the edge of the spacer jig as a guide. This insures that the tape covers approximately half of the spacer. It is important that enough of the spacer remains exposed to allow the cover glass with the mounted tissue to rest solely on the spacers and not ride up onto the tape that secures the spacers.

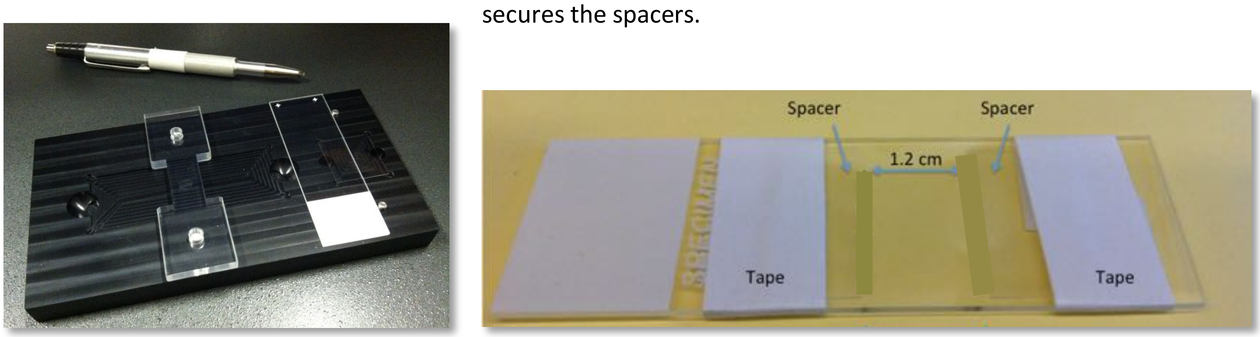

Figure S4B. Left: DPX slide making jig and diamond cleaver. Right: Slide prepared for DPX mounting.

Pre-embedding Fixation

Adult tissue to be mounted in dibutyl phthalate in xylene (DPX) requires a second fixation prior to dehydration after staining is complete, improving tissue morphology as it shrinks during dehydration.

3. Aspirate and discard the phosphate buffered saline with 0.5% Triton X-100 (PBT). Add 1.75 mL 4% paraformaldehyde (PFA) in phosphate buffered saline (PBS) and incubate for 4 hours at RT while nutating and protected from light. After the 4-hour fixation, aspirate and discard the fix.
4. Add 1.75 mL of PBT and nutate for 15 minutes at RT. Repeat for a total of 4 X 15-minute washes with PBT at RT while nutating. Always protect the samples from light.
5. If mounting does not immediately follow the 4% fixation, store the tissue at 4°C in 2 mL Protein LoBind Tubes with 1.75 PBT for up to 3 days. Nutate the tubes or lay the tubes flat in an empty freezer box while on a rotator.

Mounting

6. Wash the tissue in PBS to remove the detergent. Aspirate the PBT solution and add ~1.75 mL PBS at RT. Wash for 10 minutes on a nutator while protected from light. The presence of detergent interferes with the ability of the tissue to stick to the PLL-coated cover glass.

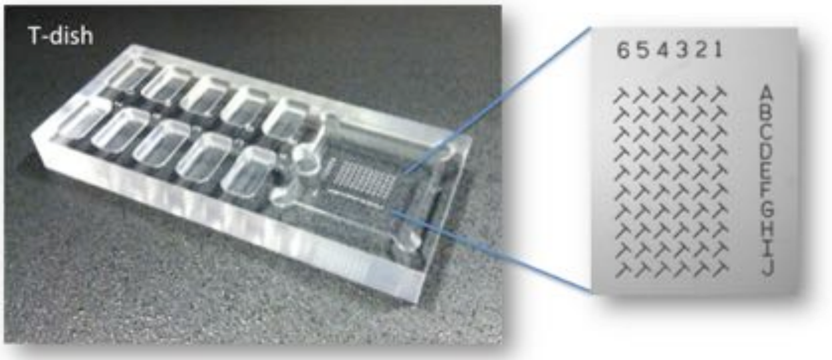

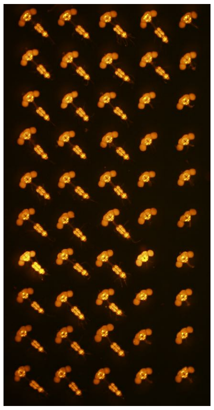

Figure S4C.

Left Top: *Drosophila* Mounting T-dish.

Right Top: Fluorescent image showing evenly spaced CNS and brains mounted using the T-dish.

Right Bottom: T-dish with cover glass and ready tissue ready to mount

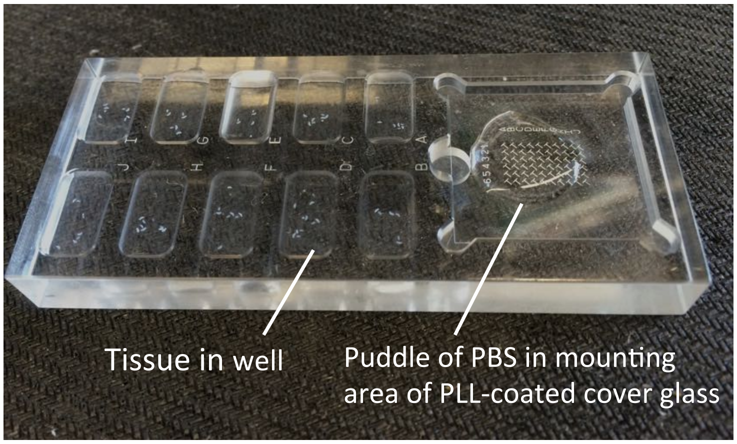

7. Add clean PBS to each well on the T-dish (Figure S4C). Do not overfill. Transfer the PBS washed tissue from a tube to a clean glass dish. Using either forceps or pipette transfer the tissues to a well on the T-dish. Avoid adding excess solution to the well during tissue transfer. The intermediate transfer step to the glass well helps avoid tissue loss from not being able to see clearly into the tube and avoids adding contaminants or debris to the mounting dish well. Each well of the T-dish holds ~120 µL.
    - The grid on the T-dish reads right-to-left (6-5-4-3-2-1), with the row IDs (A-J) on the right side. This compensates for the cover glass being "flipped" (like turning a page in a book) when the cover glass is placed "tissue down" on the prepared slide. Figure S4D.

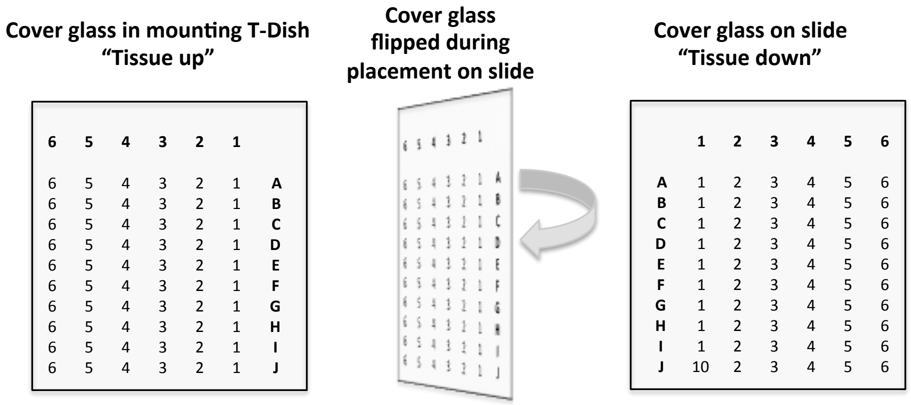

Figure S4D. Relationship between the positioning of the brains on the cover glass during mounting ("tissue up") and their position after the cover glass is flipped and placed on the slide ("tissue down").

8. Place your PLL-coated cover glass in the T-dish cover glass holder.
9. Mount brains onto the PLL-dipped cover glass in aligned columns.
    - The "T"-grid of the holder indicates how to align the tissue during mounting: the brain is placed along the top (horizontal) of the "T" and the VNC is aligned along the vertical part of the "T".
    - Add a small puddle of approximately 200 - 500 µL of fresh PBS to the region of the cover glass where you plan to place the tissue (you will see the underlying grid). Adjust the volume of the puddle to keep the samples submerged and to maintain good visibility when forceps are in the puddle of PBS.
    - Using forceps, orient the tissue anterior up in the holding well before moving it to the mounting area. Grasp the tissue in a safe place (antennal nerves, abdominal nerves or the neck connective if not handling a CNS) and transfer it quickly to the mounting area. While still holding onto the nerve, align the tissue with the "T" and gently lay it down. Do not push down on the tissue.
        - ***Hint***: It's easier to rotate the T-dish to align the "T" with the tissue rather than trying to angle the tissue to conform to the position of the "T".
10. Dip in MilliQ to remove salts. When all the tissues are mounted, remove the cover glass from the holder and dip the cover glass in a beaker of fresh MilliQ water for 2 seconds before transferring it to the first ethanol bath. If the PBS is not rinsed from the cover glass, the salts may crystalize on your tissue during dehydration and interfere with imaging.
    - Briefly touch the edge of the cover glass to the lip of the MIlliQ beaker to remove any excess water before placing the cover glass into the first ethanol bath. This minimizes diluting the ethanol.

- Make sure you maintain the orientation of the cover glass: mounted tissue facing the front and top is up. Do not rotate or flip the cover glass. Always handle the top edge of the cover glass.

Dehydration and Xylene Clearing

11. Ethanol Dehydration Series. Slowly dehydrate the tissue by sequentially submerging the cover glass in increasing concentrations of ethanol (Figure S4E). Let the tissue soak for 10 minutes in each of the following ethanol concentrations: 30%, 50%, 75%, 95% and successive soaks in three separate jars of 100% ethanol. Use a timer to precisely time each dehydration step.

Figure S4E. Cover glass staining jar filled with ethanol for dehydrating brains mounted on a cover glass. To maintain the orientation of the cover glass the front of the jar is marked in red.

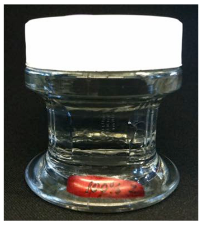

- *The cover glass __must__ remain in the same orientation (tissue facing front and top up) throughout the dehydration series. Keep the marks that identify the front of the dehydration jars facing forward when you transfer the cover glass from one jar to the next in the series.*
- Hint: Place a sticky flag on the lid of the jar to mark the slides progression through the ethanol and xylene series.
- The three successive baths of 100% ethanol have diminishing amounts of contamination of water from earlier steps. By the third 100% ethanol bath the contamination is assumed to be negligible and the ethanol concentration is essentially 100%.
- Keep the lids tightly secured on the jars at all time to prevent changes in concentration due to evaporative loss and water vapor gain.

The rest of the procedures must be done in the hood, following all appropriate lab safety practices for dealing with hazardous chemicals. Be self-informed on the hazards and risks of handling xylene and DPX.

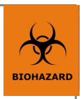

- *Always wear gloves when handling DPX and xylene.*
- *All xylene and uncured DPX must be confined to the hood*

12. Xylene Clearing. After the third 100% ethanol dehydration, submerge the cover glass in three successive baths of xylene for 5 minutes each. Use the timer in the hood to precisely time each xylene bath.
    - The cover glass __must__ remain in the same orientation (tissue facing front and top up) throughout the xylene series. Keep the marks that identify the front of the xylene jars facing forward when you transfer the cover glass from one jar to the next in the series.
    - The three baths have diminishing amounts of contamination of water and ethanol from earlier steps. By the third 100% xylene bath the contamination is assumed to be negligible and the xylene concentration is essentially 100%.
    - Keep the lids tightly secured to the jars at all time to prevent changes in concentration due to evaporative loss.

While you are waiting during the xylene baths (or earlier in the dehydration series):

- Preload the DPX dropper by lifting the tip out of the DPX and squeezing the bulb letting any DPX run down the inside wall of the bottle. While still squeezing the bulb, submerge the tip into the DPX and release the bulb to allow the tip to fill with DPX. Let the filled dropper sit before use to allow any air bubbles to rise to the top. If the dropper is squeezed while the tip is in the DPX, air bubbles may be introduced to the DPX.
- Place your slide on the mounting platform in the hood and get a blank slide (supply kept in the hood) ready to use for pressing down on the cover glass in a later step.

13. DPX application. When the last xylene soak is completed, remove the cover glass from the xylene jar (while maintaining the known orientation of tissue-to-the-front and top-up) and firmly hold it between your thumb and index finger such that the surface with the tissue is horizontal. Immediately add 7±1 drops of DPX on top of the tissue. The tissue should become transparent. (Figure S4F)
    - Xylene is highly volatile and the tissue can dry out *very* quickly. Add DPX promptly to the tissue to prevent it from drying out.
    - Wait approximately ~30 seconds to let the DPX self-level and spread around the cover glass. Hold the cover glass *as level as possible* to prevent the DPX from flowing off. Do not tilt the cover glass to speed distributing the DPX. This is known to cause problems in subsequent steps.
        - To avoid adding DPX to the wrong side of the cover glass, it is very important to make sure you maintain the orientation of the cover glass with the mounted tissue always facing the front and top is always up. *Do not rotate or flip the cover glass*.

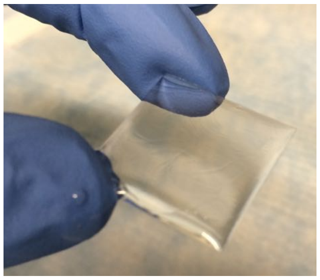

Figure S4F. DPX covering tissue mounted on a PLL-coated cover glass.

14. Position the cover glass (Slap-down: Flip-Place-Nudge-Press). Flip the cover glass over and place it – tissue/DPX down – on top of the prepared slide centered between the spacers.
    - Let the slide sit undisturbed for ~30 seconds to let the DPX spread out. Carefully nudge the cover glass with the edge of a glass slide to center and square it between the spacers.
    - Use the clean tip of a glass slide to *gently* press down <u>once</u> in the middle of the cover glass (Figure S4G). Avoid anything that could cause the cover glass to be tilted.

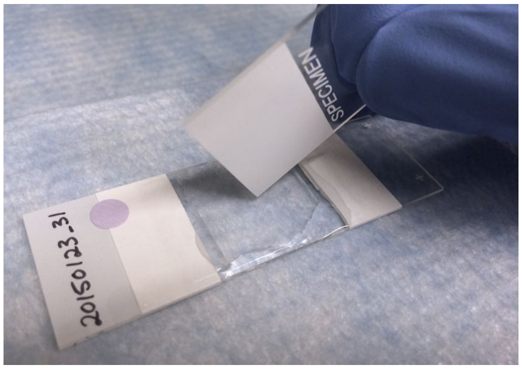

Figure S4G. Pressing down the cover glass with a corner of a glass slide.

15. Drying. Move the slide to a drying rack being careful not to tilt the slide as you move it. Cover with a cryo/freezer box lid. Let the slide dry in the hood for a minimum of 24 hours undisturbed. After 24 hours the slides can be removed from the hood, but should not be imaged until DPX is cured (approximately 2-3 days).
    - If needed, use the pointed end of a snapped cotton-tipped applicator to add a very small amount of DPX precisely where you need it (e.g. exposed edge of cover glass).
    - Excess DPX that enters the imaging area must be delicately removed before imaging. To remove excess DPX carefully nudge the tip of a scalpel under the edge of the dried DPX and fold it back. Do **not** trim the excess DPX by slicing or cutting the DPX.
    - Fingernail polish should *never* be used in attempt to seal a cut edge of cured DPX. While nail polish binds to glass it dissolves cured DPX. Tacky DPX can interact with immersion fluids and damage objectives.

- Do not use ethanol on the DPX as it will destroy the integrity of the seal. Extreme caution must be taken to avoid cracking the cover glass, breaking the seal of uncut DPX, introducing air and lifting the cover glass.

Caring For DPX Slides

1. Immersion fluids used with many high magnification objectives contain nonpolar organic compounds, which can dissolve DPX and together damage objectives. Thus, immersion fluids (e.g. Immersol 518) must be cleaned from the slide after viewing.
2. Slides made of non-aqueous mounting media such as DPX should be stored at room temperature and protected from light. Do not refrigerate or freeze.

Maintaining Solutions

1. *Ethanol and Xylene*. All series solutions are replaced (not replenished) based on the number of slides mounted. Replace cloudy solutions immediately.
2. *PLL*. Make fresh PLL as needed. Shelf life for PLL solution is 3 months when stored at 4°C. Discard immediately if turbid. Store lyophilized power at -20°C.

Safety Precautions

Xylene (dimethyl benzene) is hazardous.

- Always wear nitrile gloves when handling xylene.
- Xylene is volatile and must be stored and used in the hood only.
- For the complete MSDS for xylene see: http://www.fishersci.com/ecomm/servlet/msdsproxy?productName=X5500&productDescription=XYLENES+CERT+ACS+500ML&catNo=X5-500&vendorId=VN00033897&storeId=10652

DPX (dibutyl phthalate in xylene) is moderately hazardous.

- Always wear nitrile gloves when handling DPX.
- The xylene is volatile so uncured DPX must be stored and used in the hood only.
- Phthalates found in DPX are suspected endocrine disruptors.
- For the complete MSDS for DPX see: http://www.sigmaaldrich.com/MSDS/MSDS/DisplayMSDSPage.do?country=US&language=en&productNumber=06522&brand=SIGMA&PageToGoToURL=http%3A%2F%2Fwww.sigmaaldrich.com%2Fcatalog%2Fproduct%2Fsigma%2F06522%3Flang%3Den

Reagents and Supplies

- Corning dropping bottle (125 mL) with bulb and pipet. Fisher Scientific. # 02-986C
- Cover glass 22x22 mm Square No. 1. Fisher Scientific. # 12-542B (for mounting)
- Cover glass 22x22 mm Square No. 2. Fisher Scientific. # 12-540B (for spacers)
- Cover glass staining jar. Electron Microscopy Sciences. # 72242-24 (4 pack)
- Diamond Cleaver. Diatome.com. # CLE
- DPX Mountant for Microscopy. Electron Microscopy Sciences. # 13512, 500 mL
- DPX Slide Jig. Available through Janelia Instrument Design & Fabrication
    - http://hhmi.flintbox.com/public/project/26605
- *Drosophila* Mounting T-dish. Available through Janelia Instrument Design & Fabrication.
    - designs available: 6x10 T-dish (for adult CNS), 8x10 (for adult brains) and larval T-dish.
    - http://hhmi.flintbox.com/public/project/26606
- Ethanol. ACS reagent, >99.5% (200 proof). Sigma Aldrich. # 459844-1L
- Kodak Photo-Flo 200 Solution. Electron Microscopy Sciences. # 74257
- PBS – Phosphate Buffered Saline, 1X. Cellgro. # 21-040
- PFA – Paraformaldehyde, 20% PFA. Electron Microscopy Sciences. # 15713-S
- Poly-L-Lysine. Sigma Aldrich. # P1524-25MG
- Superfrost Plus Slides 25x75 mm. Fisher Scientific. #12-550-15
- Tape – 0.5 in. wide. Fisher Scientific. # 15-901-15A
- Triton X-100. Sigma Aldrich. # X100
- Xylenes. Fisher Scientific. # X5-500
